# Artificial intelligence for early detection of lung cancer in GPs’ clinical notes: a retrospective observational cohort study

**DOI:** 10.3399/BJGP.2023.0489

**Published:** 2025-04-23

**Authors:** Martijn C Schut, Torec T Luik, Iacopo Vagliano, Miguel Rios, Charles W Helsper, Kristel M van Asselt, Niek de Wit, Ameen Abu-Hanna, Henk CPM van Weert

**Affiliations:** Department of Laboratory Medicine, Amsterdam University Medical Center (UMC) Vrije Universiteit Amsterdam, Amsterdam; Amsterdam Public Health, Amsterdam UMC, Amsterdam, the Netherlands.; Department of Medical Informatics, Amsterdam UMC Academic Medical Center (AMC), Amsterdam; Amsterdam Public Health, Amsterdam UMC, Amsterdam; Department of Medical Biology, Amsterdam UMC AMC, Amsterdam, the Netherlands.; Department of Medical Informatics, Amsterdam UMC AMC, Amsterdam; Amsterdam Public Health, Amsterdam UMC, Amsterdam, the Netherlands.; Department of Medical Informatics, Amsterdam UMC AMC, Amsterdam; Amsterdam Public Health, Amsterdam UMC, Amsterdam, the Netherlands; Centre for Translation Studies, University of Vienna, Vienna, Austria.; Netherlands School of Public and Occupational Health, Utrecht, the Netherlands.; Amsterdam Public Health, Amsterdam UMC, Amsterdam; Department of General Practice, Amsterdam UMC AMC, Amsterdam, The Netherlands.; Julius Centre for Health Sciences and Primary Care, University Medical Centre Utrecht, Utrecht University, Utrecht, the Netherlands.; Department of Medical Informatics, Amsterdam UMC AMC, Amsterdam; Amsterdam Public Health, Amsterdam UMC, Amsterdam, the Netherlands.; Amsterdam Public Health, Amsterdam UMC, Amsterdam; Department of General Practice, Amsterdam UMC AMC, Amsterdam, The Netherlands.

**Keywords:** early detection, general practice, lung cancer, machine learning, natural language processing, oncology

## Abstract

**Background:**

The journey of >80% of patients diagnosed with lung cancer starts in general practice. About 75% of patients are diagnosed when it is at an advanced stage (3 or 4), leading to >80% mortality within 1 year at present. The long-term data in GP records might contain hidden information that could be used for earlier case finding of patients with cancer.

**Aim:**

To develop new prediction tools that improve the risk assessment for lung cancer.

**Design and setting:**

Text analysis of electronic patient data using natural language processing and machine learning in the general practice files of four networks in the Netherlands.

**Method:**

Files of 525 526 patients were analysed, of whom 2386 were diagnosed with lung cancer. Diagnoses were validated by using the Dutch cancer registry, and both structured and free-text data were used to predict the diagnosis of lung cancer 5 months before diagnosis (4 months before referral).

**Results:**

The algorithm could facilitate earlier detection of lung cancer using routine general practice data. Discrimination, calibration, sensitivity, and specificity were established under various cut-off points of the prediction 5 months before diagnosis. Internal validation of the best model demonstrated an area under the curve of 0.88 (95% confidence interval [CI] = 0.86 to 0.89), which shrunk to 0.79 (95% CI = 0.78 to 0.80) during external validation. The desired sensitivity determines the number of patients to be referred to detect one patient with lung cancer.

**Conclusion:**

Artificial intelligence-based support enables earlier detection of lung cancer in general practice using readily available text in the patient files of GPs, but needs additional prospective clinical evaluation.

## Introduction

Earlier diagnosis is the cornerstone for improved prognosis of cancer, as evidence suggests that delay in start of therapy is associated with reduced survival.^1^ Most patients with cancer present symptomatically in general practice. Much could be gained by a timelier detection in this setting.^2^ Lung cancer belongs to the cancers with the poorest prognosis. In England, the 5-year survival is 16% and has improved only slightly during the past 40 years. Survival deteriorates with increasing stage at diagnosis from 56.6% 5-year survival at stage 1 to 2.9% at stage 4.^3^

In countries with a gate-keeping system, like the UK and the Netherlands, patients can only access specialist care after referral by their GP, or in case of emergency. In the Netherlands, 90% of patients with lung cancer are diagnosed after referral by their GP.^4^ After presenting with a symptom indicative of lung cancer, the median time to referral in Dutch general practice is 13 days, with 90% being referred within 2 months.^4^ At first sight, the referral system therefore operates adequately. But, as the symptoms indicative of lung cancer are mainly caused by late-stage disease, patients with conventional and well-known symptoms mostly present with late-stage disease and about 80% of the patients are referred when the cancer is at an advanced stage (3 or 4), resulting in more invasive therapy and a worse prognosis.^5^^,^^6^

Several new technologies hold promises to enhance earlier diagnosis in general practice using new predictors (such as biomarkers), electronic-nose technology, or free-circulating tumour DNA, but these promises have not resulted in widely applicable diagnostic tests. Besides, the predictive performance of any test will be dependent on preselection.

One of the methods to improve cancer risk selection is by using text information available in the patient records of the GP. However, earlier attempts have not shown improved performance compared with existing clinical prediction tools, probably owing to the predefined nature of the predictors.^7^^–^9 In addition to predefined diagnoses, symptoms, and other patient characteristics, the patient file in primary care contains free-text notes. Free text may contain early, not yet identified clues about cancer,^10^ particularly if methods are used that can exploit the meaning of words. This study explored the potential of natural language processing (NLP) techniques and machine learning, for improving early diagnosis of lung cancer in general practice.^8^

**Table:** How this fits in

In most cancers, the prognosis depends substantially on the stage at the start of therapy. Therefore, many methods have been developed to enhance earlier diagnosis, for example, logistic regression models, biomarkers, and electronic-nose technology (exhaled volatile organic compounds). However, as most patients are referred by their GP, who keeps life-long histories of enlisted patients, general practice files might contain hidden information that could be used for earlier case finding. An algorithm was developed to identify patients with lung cancer 4 months earlier, just by analysing their files. Contrary to other methods, all medical information available in general practice was used.

## Method

### Patients and data

In this retrospective observational cohort study, free text and structured routine primary care data extracted from four Dutch primary care academic networks were used. This database contains electronic medical records from general practice networks run by the departments of general practice of university medical centres: Amsterdam (two GP networks; period 2002–2021), Utrecht (period 1995–2021), and Groningen (1989–2021). The dataset for this analysis contains longitudinal extractions of electronic medical records from routine primary care for >1.1 million patients, with up to 30 years of follow-up.

Each of the four datasets contains de-identified patient data, including structured data like sex and year of birth and laboratory results, and coded data like diagnoses and reasons for encounter. It also contains Dutch free-text descriptions of each general practice consultation.^11^ Free-text notes were written by the GP or another practice-based healthcare professional. Free-text data were further de-identified using a customised version of the DEDUCE anonymisation tool.^12^

Based on date of birth, sex, and postal code, the primary care records of patients with lung cancer were linked to the National Cancer Registry (NCR; https://iknl.nl) records (period: 1 January 1990 and 31 December 2018), using a trusted third-party linkage procedure to comply with privacy regulations of Dutch and international law (https://gdpr.eu). The NCR is a population-based cancer registry with detailed diagnostic and therapeutic data about >98% of Dutch patients with cancer. Diagnosis and month of diagnosis were extracted from the NCR and used as the reference standard.

### Data extraction

Data were retrieved about all patients with lung cancer aged ≥40 years from 2 years before registration of the diagnosis. Supplementary Table S1 provides the steps involved in processing patient data. For patients not diagnosed with lung cancer, the authors used the same period of 2 years, but up to 1 month before the last visit to the GP. As separate predictors, age and the number of occurrences of the following symptoms (International Classification of Primary Care [ICPC] codes) were added: B02 — enlarged lymph node(s); D01 — generalised abdominal pain; P17 — nicotine dependence; T03 — reduced appetite; and T08 — weight loss. These variables were selected with backward stepwise variable elimination with the Akaike information criteria^13^^,^^14^ from known predictors for lung cancer,^15^ including: A04 — fatigue; B80 — iron-deficiency anaemia; B82 — other anaemia; D87 — dyspepsia/indigestion; L04 — chest pain; K94 — thrombophlebitis/phlebothrombosis; R02 — dyspnoea; R05 — cough; R24 — haemoptysis; and R81 — pneumonia.^16^^–^18

### Time window

As a result of privacy restrictions, the study had access to the month, and not the specific date, at which the diagnosis code was entered into the database. Therefore the mid-date of the month was used as an estimation of the diagnosis date. Earlier research has shown that in the Netherlands the median diagnostic interval for lung cancer (the time from first presentation of [on hindsight] an indicative symptom to the GP until pathologically confirmed and registered diagnosis) was 49 days, of which 21 days was the median time from referral to registration of the diagnosis in the NCR (with a median time from first consultation in the hospital to diagnosis of 8 days; time from first specialist consultation to registration of the diagnosis is registered in the NCR from 2015 onwards).^4^

The predictive performance of the models when trained on 24 months of data observed 5 months before diagnoses (4 months before referral) were analysed. The authors undertook a sensitivity pre-analysis of the performance of the model (area under the receiver-operator characteristic [AUROC]) for periods 2, 3, 4, 5, and 6 months, respectively, before diagnosis. The authors chose the period with the best AUROC, although none was statistically different from the others. Previous research was also done with a 5-month timeframe.^19^

### Model development

The authors developed two models for the prediction of lung cancer. The clinical prediction architecture of both models is shown in Supplementary Figure S1. For the first model (TO: text only), only free-text consultation notes were used. For the other model (TC: text and codes) the authors used both the GP free-text notes and the added (structured or coded) patient variables with the number of occurrences of each of the ICPC codes related to lung cancer. For both models, a language model was constructed with the phrase skip-gram (PSG) algorithm^19^ that is briefly explained in Supplementary Information S1. For model 2 (TC) the authors used both the GP free-text notes, processed as described above, and the added (structured or coded) patient variables including age, sex, and the number of occurrences of each of the ICPC codes related to lung cancer.

For both TO and TC models, a logistic regression model was used to predict the probability of lung cancer. In the TO model (top part) the logistic regression model took as input the text predictors, and in the TC model (bottom part) the predictors consisted of the text embedding as well as the coded data (age, sex, and frequency of ICPC codes). Further background information and details on the PSG neural network model and the used learning algorithms are described in Supplementary Information S2.

### Internal validation

The four datasets were combined and then the merged dataset was split randomly in outcome-stratified training (60% for model development), tuning (20% for hyperparameter tuning), and testing (20% for model evaluation) sets.

### External validation

As the data originated from four different GP networks, it was also possible to perform external model validation. Therefore, a leave-centre-out approach was adopted.^20^ The authors trained a model on data from three centres and tested the model on the fourth (left-out) dataset. To provide an overall performance measure considering each centre’s share of data, the predictions obtained for each test set were stacked to yield one dataset equal in size to the size of the four datasets combined.

### Evaluation measures

Predictive performance was measured in terms of discrimination, using:

- the AUROC curve, and
- the area under the precision-recall curve (AUPRC), which gauges the balance between the positive predictive value (PPV) and sensitivity;
- the Brier score, which is the mean squared error of the predicted probabilities; and
- calibration curves, which show how close the predicted probabilities are to the observed probabilities across the full probability range.

Higher AUROC and AUPRC values mean better performance; whereas the lower the Brier score, the higher the accuracy of the predicted probabilities. The 95% confidence intervals (CIs) of AUROC, AUPRC, and Brier scores were obtained on the predicted probabilities on the test set based on the percentile method with 1000 bootstrap samples.^20^ Furthermore, to illustrate how the prediction models behave for various levels of PPV, the authors report model performance (for both internal and external validation) for the decision thresholds associated with each PPV from 0.01 to 0.10 in terms of: sensitivity, specificity, negative predictive value (NPV), and the true/false positive/negative patient numbers. The decision threshold associated with each PPV was set in each bootstrap iteration to have fixed PPVs among the iterations.

For the development of these models and the reporting of this study, the authors followed the deep learning for natural language processing (DLNLP) framework,^21^ an extension of the standard guideline for clinical prediction models Transparent Reporting of a multivariable prediction model for Individual Prognosis or Diagnosis (TRIPOD), which is suitable for NLP models. The completed DLNLP form is available in the supplementary material (see Supplementary Box S1). The source code developed for this study is available at: https://bitbucket.org/aumc-kik/aidoc.

## Results

### Patient characteristics

After patient selection, 525 526 patients out of 1 137 489 were included, of which 2386 (0.5%) were diagnosed with lung cancer (Table 1). Supplementary Table S1 shows the number of patients included at each of the main data-processing steps. Most of the exclusion of patients who did not have lung cancer was owing to age constraints (incidence of lung cancer in patients aged <40 years was <1 in 40 000). Patients with lung cancer were older (79.6% aged between 60 and 80 years) than patients without lung cancer (45.9% aged >60 years, *n* = 239 835/523 140). Supplementary Table S1 shows the descriptive statistics of the study population and Supplementary Table S2 shows the descriptive statistics for each of the data sources (that is, the four included routine primary care databases).

**Table 1.** Descriptive statistics for merged dataset based on four routine general practice care datasets together, stratified by lung cancer diagnosis

**Characteristic**	**Patients, *n* (%)**		

	**No cancer, 523 140 (99.5)**	**Cancer, 2386 (0.5)**	**Total, 525 526 (100)**
**Gender, female, *n* (%)**	274 908 (52.5)	1100 (46.1)	276 008 (52.5)

**Age, years, *n* (%)**			
40–59	283 305 (54.2)	486 (20.4)	283 791 (54.0)
60–69	111 653 (21.3)	814 (34.1)	112 467 (21.4)
70–79	77 642 (14.8)	762 (31.9)	78 404 (14.9)
≥80	50 540 (9.7)	324 (13.6)	50 864 (9.7)

### Internal validation

Supplementary Table S3 shows the AUROC and AUPRC model performance at different timeframes before diagnosis (internal validation on 20% of patients). Predicting up to 5 months before diagnosis achieved the highest performance, for instance, the PSG-based TO model at 5 months before diagnosis yielded an AUROC of 0.88 (95% CI = 0.86 to 0.89), although results for other time windows are comparable.

**Figure 1.**
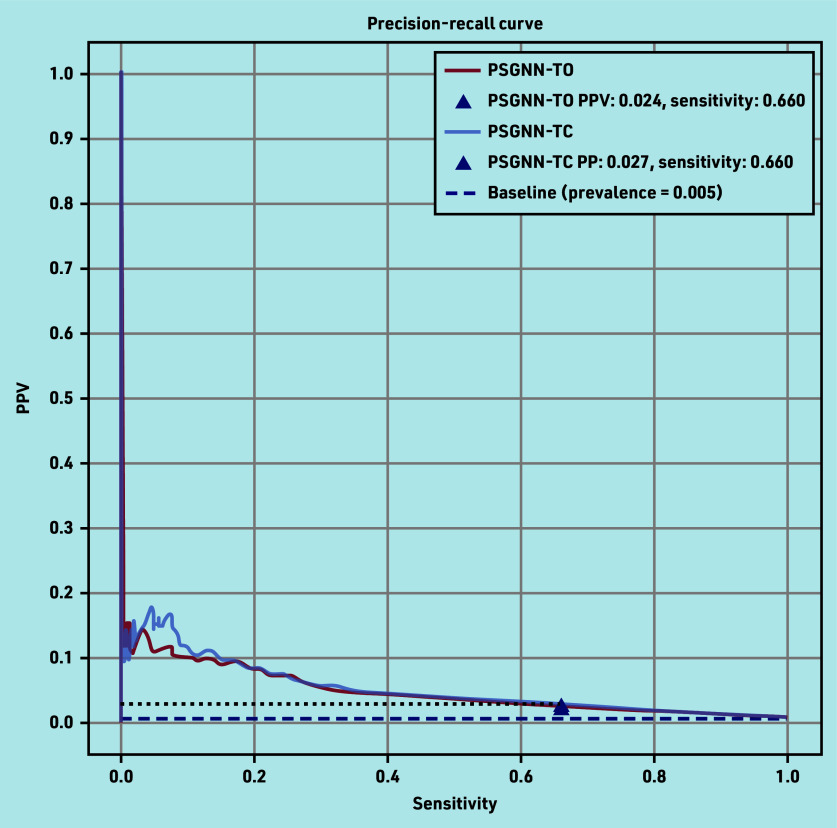
Precision-recall curves of the phrase skip-gram (PSG) model when used with text only (TO) and when used with text and coded data (TC). PPV = positive predictive value. PSGNN = phrase skip-gram neural network.

Supplementary Table S3 also shows the AUROC, AUPRC, and Brier score of the PSG-based model when used with the text-only model (TO) and when used with text and ICPC coded data (TC). Both models had excellent discriminatory performance (AUROC ≥0.87) at 5 months before diagnosis. The TC model yielded similar performance to the TO model. The precision-recall curves of both models are shown in Figure 1.

Supplementary Table S4 displays how the accuracy, specificity, sensitivity, and NPV of the PSG-based models at 5 months before diagnosis vary when using a decision threshold corresponding to different PPVs (risks). For all the measures, the TC model achieved a similar performance to the TO model.

**Figure 2.**
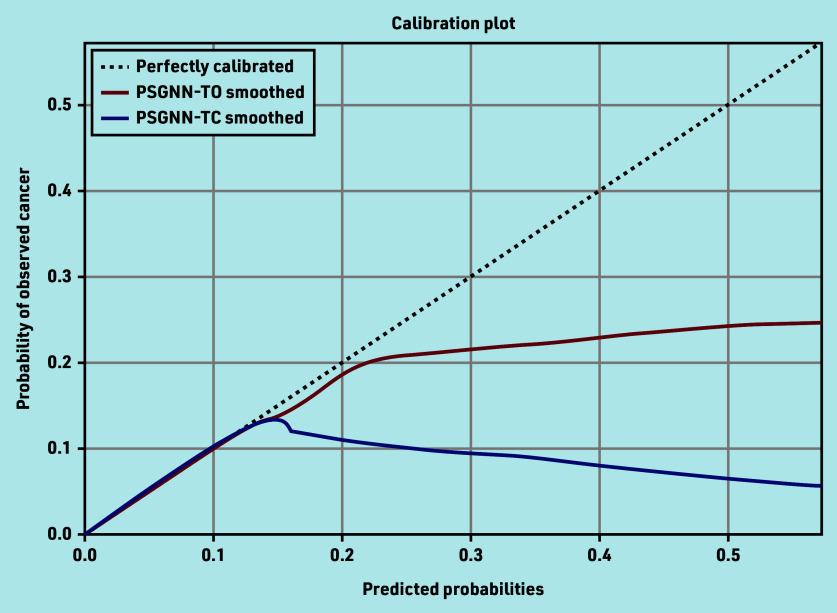
Calibration plot of the phrase skip-gram (PSG) model when used with text only (TO) and when used with text and coded data (TC). The plot shows actual probabilities (y-axis) versus predicted probabilities (x-axis), and an ideal curve (dotted line) is included for illustrating when the predicted probabilities are identical to the actual probabilities. PSGNN = phrase skip-gram neural network.

Figure 2 shows the calibration curves of the models. The two models are similarly calibrated. Despite its excellent diagnostic performance, and owing to the low prevalence of the outcome, a relatively large number of patients are falsely labelled as positive. For example, to correctly identify 291 patients with lung cancer (PPV 3%) 4 months earlier (that is, before the GP referred the patient), 185 are missed (with the TO model) and 9409 are identified as at increased risk (Supplementary Table S4, data in italics).

### External validation

Table 2 shows the AUROC, AUPRC, and Brier scores of the TO and TC models for each of the leave-one-out validation analyses. Both models had good discriminatory performance (AUROC >0.80) when using data from three out of four centres as a test set and close to good overall performance (AUROC 0.78–0.79). The models achieved similar performances. Table 2 also shows that the overall performance of the TO model (which performed best in internal validation) achieved an AUROC of 0.79 (95% CI = 0.78 to 0.80). Supplementary Table S5 shows how the accuracy, specificity, sensitivity, and NPV of the TC models vary when using a decision threshold corresponding to different PPVs. These results were obtained by stacking the predictions of each centre on top of each other.

**Table 2.** Model evaluation results for each of the leave-one-out validation analyses of the TO and TC prediction models, which used text only and both text and coded data, respectively

**Model and test set**	**AUROC (95% CI)**	**AUPRC (95% CI)**	**Brier score (95% CI)**
**TO**			
AHON-UMCG	0.7187 (0.6910 to 0.7452)	0.0154 (0.0118 to 0.0271)	0.0046 (0.0043 to 0.0050)
ANH-VUmc	0.8235 (0.8051 to 0.8417)	0.0515 (0.0387 to 0.0664)	0.0052 (0.0048 to 0.0056)
JGPN-UMCU	0.8054 (0.7908 to 0.8189)	0.0371 (0.0310 to 0.0452)	0.0049 (0.0046 to 0.0052)
AHA-AMC	0.8133 (0.7951 to 0.8312)	0.0210 (0.0169 to 0.0257)	0.0038 (0.0035 to 0.0042)
Overall	0.7875 (0.7778 to 0.7965)	0.0282 (0.0248 to 0.0324)	0.0046 (0.0044 to 0.0048)

**TC**			
AHON-UMCG	0.7167 (0.6923 to 0.7412)	0.0181 (0.0129 to 0.0271)	0.0044 (0.0041 to 0.0048)
ANH-VUmc	0.8192 (0.7997 to 0.8365)	0.0505 (0.0395 to 0.0658)	0.0051 (0.0047 to 0.0055)
JGPN-UMCU	0.8099 (0.7959 to 0.8243)	0.0421 (0.0345 to 0.0522)	0.0049 (0.0046 to 0.0052)
AHA-AMC	0.8043 (0.7855 to 0.8218)	0.0253 (0.0193 to 0.0332)	0.0038 (0.0035 to 0.0042)
Overall	0.7817 (0.7720 to 0.7906)	0.0219 (0.0195 to 0.0247)	0.0046 (0.0045 to 0.0048)

*AHA-AMC = Academic general practitioners network database, Amsterdam UMC location University of Amsterdam. AHON-UMCG = Academic General Practitioners Network Northern Netherlands database, University Medical Center Groningen, University of Groningen, Groningen, the Netherlands. ANH-VUmc = Academic Network of General Practice database, Amsterdam UMC location Vrije Universiteit, Amsterdam, the Netherlands. AUPRC = area under precision-recall curve. AUROC = area under the receiver-operator characteristic. JGPN-UMCU = Julius General Practitioner’s Network database, University Medical Centre Utrecht University, Utrecht, the Netherlands. TC = with text and coded data. TO = text only.*

## Discussion

### Summary

The results demonstrate that text analysis of routine primary care consultation notes can facilitate earlier detection of patients with lung cancer. The basic risk in the population studied (aged ≥40 years, general population enlisted in general practice) for lung cancer was 0.4%, after cleaning the dataset. In internal validation, the algorithm developed increased that risk almost tenfold (Supplementary Table S4, TO model) thus adequately referring 62% (*n* = 1480/2386) of all patients with lung cancer around 4 months earlier than present Dutch practice. Given the stage-related prognosis of lung cancer, this is a clinically relevant improvement.^1^ Earlier identification of these patients saves lives.^22^ However, it does come at a cost.

Calculated with a posterior chance of 3% for lung cancer^23^ (this is the threshold for referral as mentioned by the UK National Institute for Health and Care Excellence) for every patient diagnosed 4 months earlier, 33 patients will need to undergo diagnostic evaluation because of presumed increased risk (for example, a chest X-ray or low-dose computer tomograph scan^24^) and about 40% of all patients with lung cancer will be missed and be referred 4 months later following usual care.^23^ These numbers of course are dependent on prevalence in the population and the chosen cut-off (the current study used 3% PPV). Although, as expected, performance slightly decreased in the external validation, models preserved a good predictive performance (AUROC close to 80%). Given their similar performance, the simpler TO model can be preferred in future application compared with the more complex TC model.

The development of machine learning-based decision support systems is a promising but complex process. This is also true for its road to implementation. Besides the methodological, technical, and epidemiological challenges, ethical, societal, legal, and privacy issues should also be considered in future research. This methodology might also be helpful to detect other ‘silent’ types of cancers with a poor prognosis, such as pancreatic, oesophageal, and ovarian cancer. For development of detection support models addressing these relatively rare cancers, larger datasets than this one will be necessary, which makes international collaboration valuable and necessary.

### Strengths and limitations

This ‘proof of concept’ study has several strengths. The study used actual routine primary care data of a representative Dutch population (the authors selected patients only for the presence of data in the records of GPs and the presence of a validated diagnosis in the NCR), which makes the results valid for all patients aged ≥40 years with a general practice file including a history of at least 2 years and at least one free-text line. As in the Netherlands almost all inhabitants are enlisted with a general practice and GPs act as gatekeepers, even patients that present acutely to emergency departments or bypass the GP otherwise, are still included in the current analysis.

GPs will mostly code data when they think the data are relevant; therefore, coded data form a selection of available information and this leads without doubt not only to ‘confounding by indication’, but also to loss of information. This is more so, as Dutch GPs mostly will not code >1 symptom and will only use a symptom code when a diagnosis is lacking or not suspected. By using free text, the current study avoided this coding bias. Both internal and external validation were performed showing the consistency of the diagnostic signals in the data.

However, the authors’ approach also had limitations. International differences in data structure, patient presentation, and GP’s registration may limit the usability and applicability of clinical algorithms internationally. But, despite these limitations, the methods used in the current study can be adapted for international use, if regional-specific development and validation are performed. Further, it should be noted that the algorithm can best be used (by the GP) after adding information to the existing file (after receiving test results or integrated in a consultation), so that new information can be incorporated in the diagnostic assessment. Further, the GP should always assess the relevance of a warning signal as, of course, the medical history of a patient does not change and a relevant history might evoke a warning signal repeatedly. In addition, it should be remembered that the sensitivity of a chest X-ray is around 75%. Also, when used with a predictive power of 3%, the algorithm misses four out of 10 patients.

Another limitation comes from the current study’s case ascertainment procedure where the authors could only indirectly match GP patients to the cancer registry, where some patients with cancer may not be identified because they moved between postal codes during the period under analysis. And one of the limitations of neural networks for text analysis is that the outcome is a ‘black box’; it is not known which texts, words, or phrases do determine the increased risk for lung cancer. If implemented in daily practice, it might be challenging to convince GPs and patients of the validity of the process. Related to this limitation, a general word of caution for evaluating clinical prediction models is that *‘prediction performance is not the only factor to consider when evaluating models for healthcare applications’*.^25^ Further research is needed to overcome these limitations.

A large database was used in the current study and similar performances were achieved at different timeframes before diagnosis and by the TO and TC models (CIs in the performance metrics overlapped). However, the authors recommend replicating the current study with a larger database and in different healthcare systems, which might need international collaboration.

### Comparison with existing literature

To the authors’ knowledge there are no similar studies. Many methods have been developed to enhance earlier diagnosis: for example, logistic regression models, biomarkers, and electronic-nose technology (exhaled volatile organic compounds). However, as most patients are referred by their GP, who keeps life-long histories of enlisted patients, general practice files might contain hidden information, which could be used for earlier case finding. The authors developed an algorithm to identify patients with lung cancer 4 months earlier, just by analysing their files. Contrary to other methods that, for example, only use pre-defined variables,^7^^–^9 all medical information available in general practice was used.

### Implications for practice

The outcome of the current research is not to be compared with screening: the timeframe is much shorter, the positive prediction is much higher, and it does not use any diagnostic procedures, just an algorithm, that can be integrated in a practice-based registration system. Compared with previous studies, the current prediction model is more accurate and more flexible (adaptable to local situations).^9^ The algorithm can be fine-tuned to fit several settings, each with its own implications for clinicians and policymakers. The study analysed PPVs of 1% to 10%, but these can be adopted as desired, as sensitivity and specificity of the algorithm can be adapted to the impact of the disease that it is aiming to find and/or to the follow-up examinations that will be needed. For primary care practice, the current study has produced a model able to indicate a relevant risk of lung cancer several months earlier, with a potentially significant impact on prognosis.^1^ For primary care clinicians, the result might be assistance to achieve their main objective — earlier recognition of a substantial risk of serious disease. When calculated with a prevalence of 3% among 1000 referred patients, the number of correctly identified patients with a chest X-ray will be 25, and five will be missed, while minimising the risk of overdiagnosing.^24^

## Data

Data cannot be shared publicly because of patient confidentiality. Data are available from the research networks (AHA-AMC, Academic general practitioners network database, Amsterdam UMC location University of Amsterdam; AHON-UMCG, Academic General Practitioners Network Northern Netherlands database, University Medical Center Groningen, University of Groningen, Groningen, the Netherlands; JGPN-UMCU, Julius General Practitioner’s Network database, University Medical Centre Utrecht University, Utrecht, the Netherlands; and ANH-VUmc, Academic Network of General Practice database, Amsterdam UMC location Vrije Universiteit, Amsterdam, the Netherlands) for researchers who meet the criteria for access to confidential data.
